# Botulinum toxin improved intestinal adaptation to short gut in a twenty-one-day-old weanling rat

**DOI:** 10.1590/1414-431X2024e14124

**Published:** 2025-01-31

**Authors:** W.C. Canesin, F.P. Volpe, L. Falquetti, M.Q. Marques, I.C.S. Marques, R.S. Saia, R. Gadde, S.B. Garcia, L. Sbragia

**Affiliations:** 1 Divisão de Cirurgia e Anatomia Pediátrica, Departamento de Cirurgia e Anatomia, Faculdade de Medicina de Ribeirão Preto, Universidade de São Paulo, Ribeirão Preto, SP, Brasil; 2 Departamento de Patologia, Faculdade de Medicina de Ribeirão Preto, Universidade de São Paulo, Ribeirão Preto, SP, Brasil; 3 Departamento de Fisiologia, Faculdade de Medicina de Ribeirão Preto, Universidade de São Paulo, Ribeirão Preto, SP, Brasil; 4 Division of Pediatric Surgery, Nationwide Children's Hospital, Columbus, OH, USA

**Keywords:** Necrotizing enterocolitis, Short gut, Botox, Experimental model, Adaptation

## Abstract

Necrotizing enterocolitis (NEC) is a severe intestinal disease of multifactorial origin that primarily affects premature infants. Approximately 27% of NEC babies develop short gut (SG) secondary to extensive intestinal resection, and 10% will have chronic dependence on total parenteral nutrition. We evaluated the Botox treatment in SG model rats. Twenty-day-old weanling male rats (weight range 38-70 g, n=72) were divided into four groups (n=18 each): 1) Control (fed a regular liquid diet); 2) Botox (Control submitted to laparotomy and intestinal injection of Botox^®^); 3) SG (short gut); and 4) SG and Botox (SG+Botox^®^). After seven post-operative days, samples were collected for biometrics [body weight (BW), intestine weight (IW) and IW/BW ratio (IBR), and intestine length (IL) and height (IH)], histometric analysis [villous height (VH), crypt depth (CD), muscular thickness (MT), and PCNA index)], and intestinal transit time (ITT). BW, IW, and IL decreased in SG (P<0.05). IH, VH, and PCNA index increased in Botox groups [Control = SG < Botox and SG+Botox (P<0.05)], CD increased in Botox, SG, and SG+Botox (P<0.005), and MT was higher in SG and SG+Botox. Botox groups had lower ITT (P<0.05). Botox provided dilatation and histological changes in SG. These findings suggested that Botox improved adaptation and might be applied in SG with promising results.

## Introduction

Short bowel syndrome (SBS) is defined as a malabsorptive state resulting from extensive intestinal resection or dependence on prolonged total parenteral nutrition (TPN) (>90 days) secondary to intestinal failure. In children, intestinal autonomy is possible when the remaining intestine is ≥60 cm in the absence of the ileocecal valve (ICV) or ≥40 cm in the presence of ICV (1,2). Intestinal failure, in turn, is characterized by the loss or decrease of intestinal absorptive capacity to maintain nutritional status (growth, hydration, and electrolyte balance) exclusively through the enteral route (3). The most frequent causes of SBS in infants include necrotizing enterocolitis (NEC), complicated meconium ileus, gastroschisis, intestinal atresia, and volvulus (4). Dependence on TPN causes complications such as liver failure, central venous catheter-related infections, and exhaustion of venous access, which are the leading causes of death among these patients. The average survival time of TPN-dependent children is 1-2 years. Additionally, TPN serves as a bridge between definitive treatment modalities with intestinal and liver transplantation resulting from prolonged TPN and recurrent sepsis (5). Approximately 12-15% of childhood intestinal transplants are secondary to NEC (6). The development of new therapies that aim to improve intestinal adaptation after extensive resection is needed to improve and save the lives of children with SBS (7). Several autologous intestinal reconstruction procedures have been described to increase the functional absorptive area of the intestines and facilitate intestinal motility while limiting stasis (8,9). The use of botulinum toxin as an adjunct to the surgical treatment of diseases that affect the gastrointestinal tract, such as achalasia, anal fistula, and Hirschsprung's disease, is well established in children (10). In disorders that affect the anal sphincter, such as obstructive defecation, its application can promote reversible relaxation of the muscles adjacent to the anus without causing fecal incontinence (11). We have recently observed good weight gain with the use of the toxin in the SG model in adult rats (12). Therefore, our objective was to evaluate the local applicability of Botox as a strategy for intestinal adaptation in SBS in recently weaned rats for possible application in children.

## Material and Methods

All procedures followed the guidelines of the National Council for the Control of Animal Experimentation (CONCEA, Brazil) and were approved by the Institutional Animal Care and Use Committee of Ribeirão Preto Medical School, University of São Paulo (CEUA protocol number 0053/2020).

### Animals

Male Wistar rats (Central Vivarium, Ribeirão Medical School, University of São Paulo) were weaned at 21 days of age (weight of approximately 40 g). The animals were placed in standard cages and maintained on a 12-h light/dark cycle at an ambient temperature of 25±1°C. The animals were separated from their mothers, one day before the experiment, *ad libitum* access to water was provided until the night before the experiment; the animals were fasted for 12 h before surgery. The following day, the rats underwent surgery in the morning. After the animals recovered from surgery, they were fed a specific Lieber-DeCarli liquid diet for seven days (from the first to the seventh postoperative day). Control animals were subjected to the same protocol, except for surgery. This diet contains 1.0 kcal/mL, of which 35% is from fat, 47% from carbohydrates, and 18% from proteins, providing 820 non-protein kcal/L and 180 protein kcal/L, totaling 221.78 g of powder for 1 L of liquid diet. The powdered content of the diet was dissolved in cold filtered water until it reached 1 L and blended in a blender at low speed for 30 s. Once ready, the diet lasted for 48 h at room temperature; after that, all content was discarded and replaced with a new diet.

### Study groups

Four groups were studied: a) Control: fed a regular liquid diet without surgical intervention (n=18); Botox (Control+Botox^®^), submitted to laparotomy and intramuscular injection of 20 U of Botox^®^ in the intestinal wall (n=18); SG (short gut), submitted to laparotomy and resection of the loops in a short intestine model (n=20); and SG+Botox (short gut and Botox^®^), submitted to laparotomy and resection of the loops in a model of short intestine with intramuscular injection of 20 U of Botox^®^ in the intestinal wall (n=23).

### Surgical technique for making the short intestine

The rats were submitted to intramuscular sedation with ketamine hydrochloride (Ketamina^®^, Pfizer, Brazil) in the quadriceps of the thigh followed by local anesthesia in the midline (incision) with Lidocaine Hydrochloride (Xylestesin^®^, Brazil) with 1% vasoconstrictor diluted in 0.9% NaCl making the solution 0.5% and applied 1 mg/kg on the skin and subcutaneously with a 22G needle and subsequently with an intraperitoneal injection of sodium thiopental (Thiopentax^®^, Brazil) 30 mg/kg. This anesthetic composition kept the animal under deep anesthesia for two hours, and the postoperative period was painless during the immediate period.

**Figure 1:**
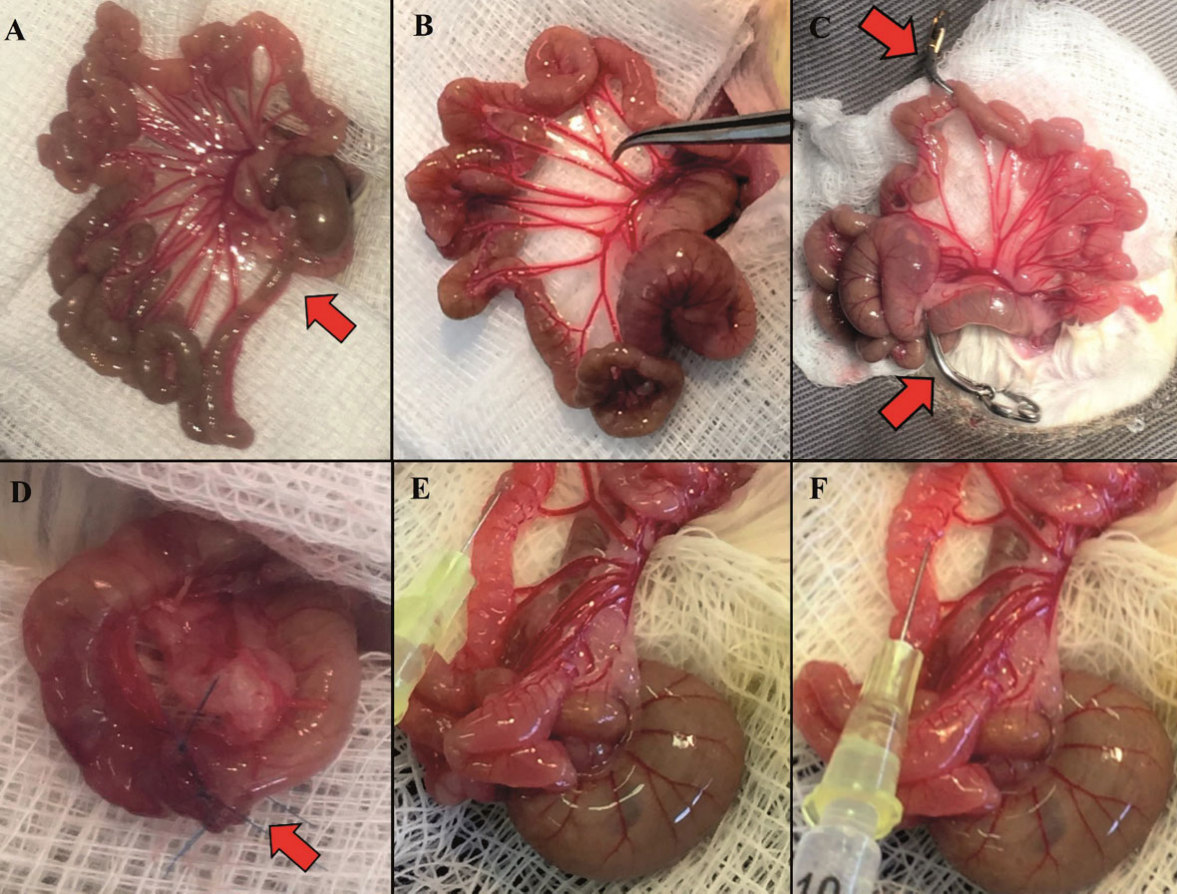
Steps of the surgical procedure. **A**, Ileocecal valve (arrow); **B**, Intestinal arcades (approximately 5-6); **C**, Amount of intestinal resection between clamps; **D**, Anastomosis (arrow); **E** and **F**, Botox application in the intestinal borders.

The surgical procedure for the short gut (SG) was performed according to Yang et al. (13). The abdominal region was shaved, taking care not to injure the skin. After asepsis with chlorhexidine aqueous solution and placement of sterile drapes, the animal underwent median laparotomy with an incision of around 2.5 cm. The ileocecal valve and the colon were identified, the intestinal arcades were counted, and the length of the intestine removed was always located between the fifth and sixth distal mesenteric arcade from the cecum. The intestine was resected after ligation of the mesenteric vessels, resulting in the removal of an approximate average of 30 cm of small intestine. The ileum, ileocecal valve, cecum, and 1 cm of ascending colon were removed. The end-to-end anastomosis of the jejunum and the proximal right colon was performed using six separate sutures with Blue Polypropylene 7.0 (Prolene^®^ Ethicon Inc., USA). In the SG+Botox group, after intestinal resection, using a 25G needle, 20U of Botox^®^ was applied (intramuscular injection of the intestinal wall) 1 cm above the anastomosis, with 10 U each on the mesenteric and the anti-mesenteric edge. This was then followed by closure from the wall using two layers. The Botox Control underwent laparotomy with manipulation of the intestinal loops, counting the 5 or 6 arches from the ileocecal valve and injecting Botox^®^ in the same manner. To identify the injection site in the future harvest, a suture was made in the mesentery with the same thread used for the anastomosis in the SG group. After the anastomosis, 1 mL of 0.9% saline solution was added to the abdominal cavity. The cavity closure was performed by planes with Polyglactin 4.0 sutures (Vicryl^®^, Ethicon Inc., USA) with continuous stitches and by skin with Nylon 5.0 sutures (Ethicon Inc.) with continuous intradermal stitches. The postoperative period occurred in acrylic cages, in individual pens, with the same liquid diet and water offered ad libitum (Figure 1).

### Time of intestinal transit

On the seventh postoperative day, with n=8 animals per group, gavage was performed with dextran-TRITC 40 kDa (100 µL of 20 mg/mL solution diluted in PBS, Sigma-Aldrich, USA) in each animal. After 45 min of waiting for the marker to spread throughout the gastrointestinal tract (GIT), rats were anesthetized, and the entire GIT was collected for analysis. After removal, segments were gently placed on the surface of a paper towel to determine the total length of the respective intestinal segment and the patency of the anastomosis in the specific groups, where the tweezers were passed through the lumen of the intestinal loop. The segments were divided into nine parts from the stomach to the rectum and maintained in tubes containing PBS, being identified as S: stomach; PJ: proximal jejunum; MJ: middle jejunum, DJ: distal jejunum; JIT: jejunum ileum transition; PI: proximal ileum; DI: distal ileum; PC: proximal colon; and DC: distal colon. Intestinal transit time (ITT) was evaluated after the anti-mesenteric edge of the GIT segments was opened, shaking the Falcon tubes in the vortex for 1 min, then centrifugation at 12,298 g for 5 min at 36.5°C. Fluorescence intensity was quantified in the supernatant by spectrofluorimetric method in black 96-well plates, excitation wavelength = 550 nm, and emission wavelength = 577 nm (14). Data are reported as a percentage of the marker in each segment compared to the total amount of the fluorescent marker present in the GIT of each rat.

### Harvest

On the seventh day after intestinal resection, the animals were anesthetized again in the same manner and with the exact dosages of drugs with which they were previously operated. The animal was weighed, the abdomen was opened, and the intestine was removed (from the duodenum to the rectum), weighed, and immediately fixed in a suitable solution for subsequent histological analysis. Then, still under anesthesia, the animal was sacrificed by exsanguination by opening the two diaphragm muscles and opening the left heart ventricle with 14 cm surgical scissors. Also, on the seventh day after resection, the animals that underwent intestinal transit studies (n=8 per group) had their intestinal samples collected exclusively for this purpose and were not saved for histology. After studying intestinal transit, the animals were anesthetized and sacrificed in the same way as the others.

### Morphometric evaluation

Body weight (BW), body weight on day 7 (BW), liver weight (LW), intestine-to-body weight ratio (IW/BW ratio), intestine weight (IW), intestine length (IL), and intestine height (IH) were measured daily.

### Histological evaluation

Intestinal tissue samples were collected from each animal 1 cm above the anastomosis in the resection groups and Botox group at the marked site of the remaining jejunum/ileum, covering the entire thickness of the small bowel wall for microscopic evaluation. Samples were immediately fixed in buffered formalin (pH 7.4) for 24 h, dehydrated, and then embedded in paraffin and transversely sectioned into 5-μm-thick slices using a Leica microtome (Model RM214; Leica, Austria). Four sections were obtained per animal for 5 animals per group, stained with hematoxylin and eosin (H&E), and used for the histometric study of the intestinal wall. The following histological parameters were evaluated: villus height (VH), crypt depth (CD), muscle lamina thickness (MT), and PCNA index to establish objective criteria for intestinal adaptation in the period, with and without intestinal application of Botox^®^. Immunohistochemistry was used to measure and evaluate PCNA, which has nuclear labeling. Specifically, we utilized a quantitative method, counting immunohistochemically labeled cells per 1,000 cells counted on the slide, taking care to count cells in the four quadrants of each slide. Based on this calculation, we obtained the cell proliferation index (number of PCNA-labeled cells per 1,000 cells) for the intestinal area (jejunum) where Botox was applied. The height of the villi and the depth of the crypts were calculated as the average of 10 villi and 10 crypts and are reported in μm. The evaluation was performed in an Olympus light microscope CX 41 (Japan) at 40× magnification using an ocular micrometer. The images obtained from an Olympus DP 20 high-definition photo receiver coupled to a light microscope were interpreted.

### Statistical analysis

Frequency tables for categorical variables and descriptive statistics (with measures of position and dispersion) for continuous variables were prepared to describe the sample profile according to the variables under study. Data were evaluated using the Kolmogorov-Smirnov test to assess evidence that they could have a normal distribution. Analysis of variance (ANOVA) and Tukey's post hoc test for repeated measures were used to compare variables between groups and gestational ages. The GraphPad Prism version 9.2 for Windows program (GraphPad Software, USA) was used. Statistical significance was considered when P values <0.05.

## Results

**Figure 2:**
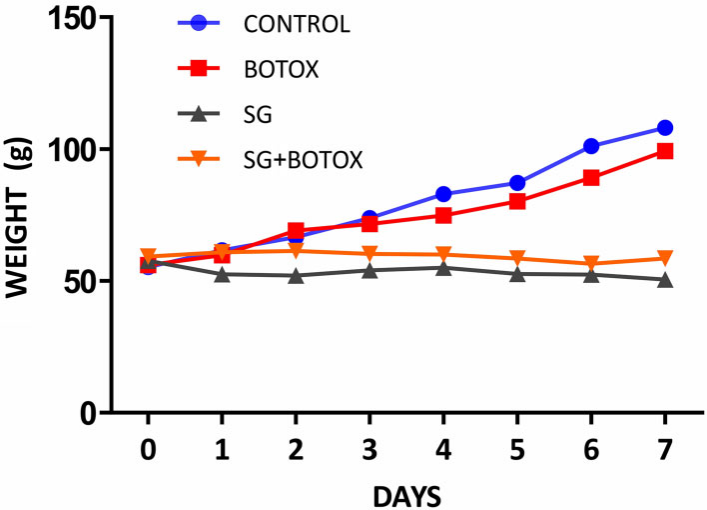
Daily total weight from day 0 to day 7 of the procedure of all experimental groups. There was no statistical difference between Control and Botox or between SG and SG+Botox.

In totality, there were 79 infant rats, of which 72 were included in the study. Mortality varied among the groups, with deaths exclusively in the surgical group. The Control and Botox groups had no deaths (n=36), however the SG and SG+Botox groups had 7 deaths (n=43). The SG group had 10% of deaths (2 out of 20 = 18 survivals), and the SG+Botox group had the highest proportion of deaths, 23% (5 out of 23 = 18 survivals). In the SG+Botox group, there were five deaths due to post-operative complications; four had anastomotic leakage with peritonitis, and one had anastomotic site stenosis. In the SG group, there were two deaths, and the cause of death could not be identified accurately. For all four groups, the total daily weight from day 0 to day 7 of the procedure is graphically represented in Figure 2.

### Morphometric analysis

**Figure 3:**
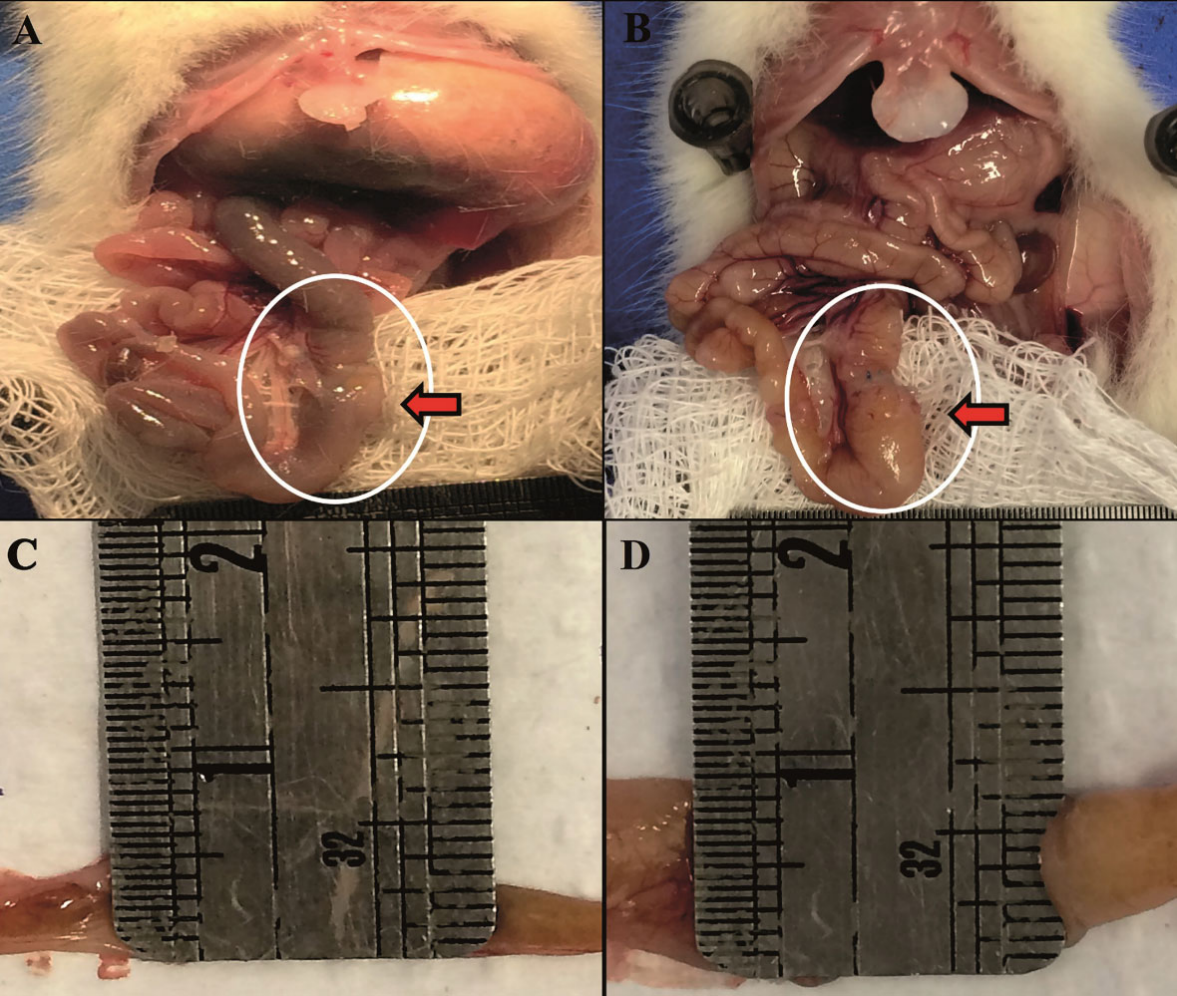
Location of injected Botox and measurement of intestinal height at day 7. **A**. Botox; **B**, SG+Botox; **C**, normal intestinal height; and **D**, intestinal height with Botox. SG: short gut.

The results of the morphometric analysis are shown in Table 1 and Figure 3. There were no differences in intestinal length between Control vs Botox or SG vs SG+Botox. However, we found a difference between Control and Botox (n=36) vs SG and SG+Botox (n=36) (P<0.005).

**Table 1:** Morphometric results among the four experimental groups.

	Control	Botox	SG	SG+Botox
Body weight on day 7 (g)	101.09 (±6.95)	106.03 (±11.77)	53.61 (±8.60)	51.11 (±10.74)^c^
Liver weight (g)	4.91 (±0.32)	5.13 (±0.73)	2.61 (±0.61)	2.57 (±0.60)^c^
Intestine weight (g)	14.64 (±2.22)	14.55 (±1.57)	6.13 (±2.17)	4.19 (±1.37)^c^
Intestine to body weight ratio (%)	0.1415 (±0.011)	0.1397 (±0.0185)	0.1029 (±0.0245)^b,c^	0.0768 (±0.0077)^c^
Intestine length (mm)	152.00 (±12.67)	149.20 (±9.69)	58.92 (±22.89)^b,c^	43.54 (±14.42)^c^
Intestine height (mm)	4.50 (±0.54)	7.66 (±1.23)^a^	5.00 (±0.89)^b,c^	9.37 (±1.06)^c^

^a^Botox *vs* Control (P<0.005; ANOVA and Tukey test), ^b^SG *vs* SG + Botox (P<0.005, ANOVA and Tukey test), ^c^SG and SG + Botox *vs* Control and Botox (P<0.005; ANOVA and Tukey test).

### Histology

**Figure 4:**
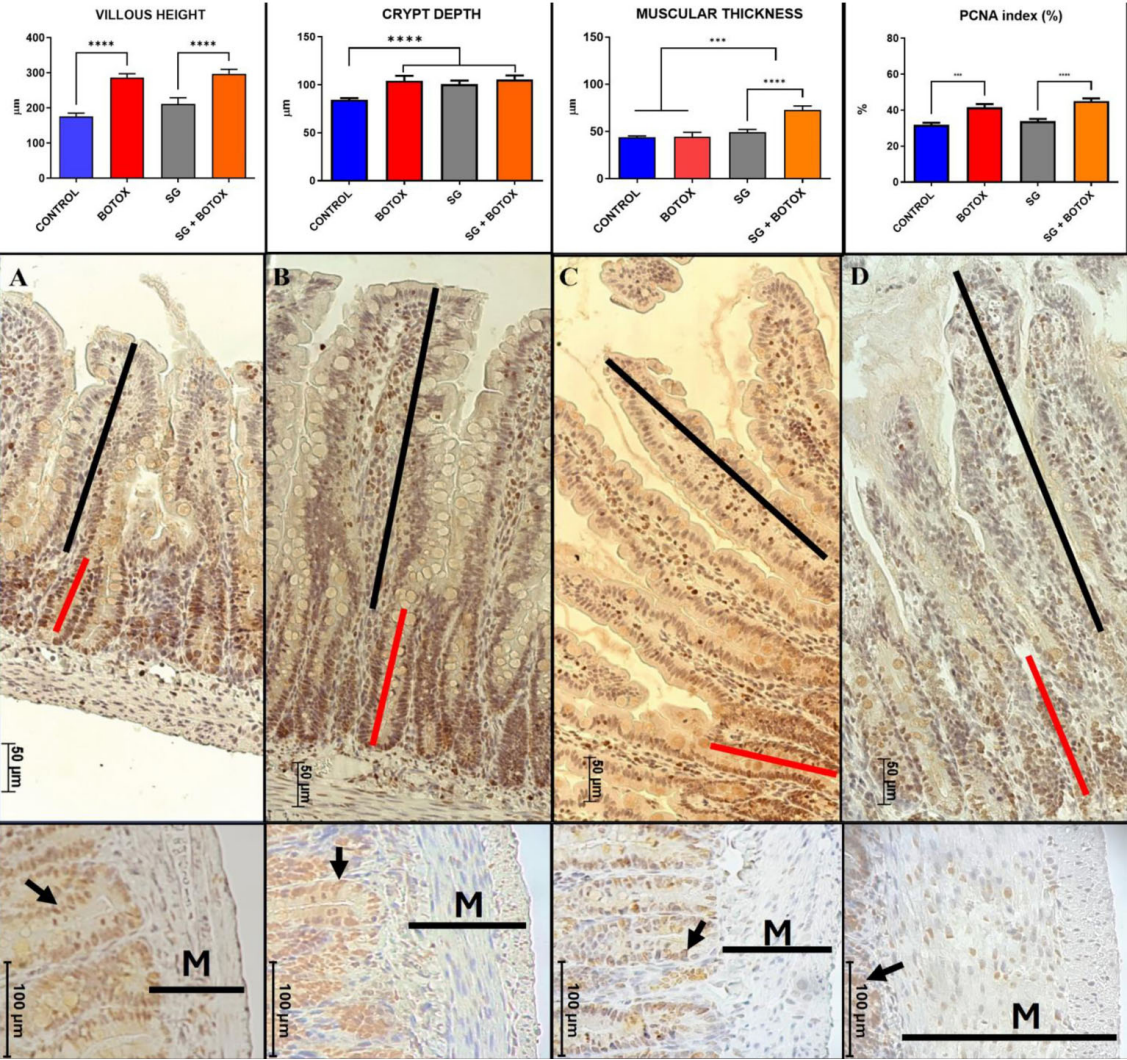
Histometric results including grouped analysis amongst the four experimental groups. ***P<0.0005, ****P<0.0001 (ANOVA and Tukey's *post hoc* test). Photomicrographs of the small intestinal villi for the four experimental groups: **A**, Control; **B**, Botox; **C**, SG; and **D**, SG+Botox. Black bars: villous height, red bars: crypt depth. Bottom panels: black bars: length of the muscle layer (M) of the gut wall, arrows: PCNA-positively stained nuclei of epithelial cells. Scale bars: middle panels 50 μm and bottom panels 100 μm.

Villus Height: Control 175.8 μm (±9.48), Botox 286.3 μm (±10.81), SG 210.8 μm (±18.13), and SG+Botox 297.0 μm (±12.77). There were significant differences between Control vs Botox (P<0.005) and SG vs SG+Botox (P<0.005). There was no significant difference between Control vs SG or Botox vs SG+Botox. Botox increased the villus height. Crypt Depth: Control 84.50 μm (±1.69), Botox 104.3 μm (±5.17), SG 100.6 μm (±3.85), and SG+Botox 105.4 μm (±4.43). There was a difference between Control vs Botox, SG, and SG+Botox (P<0.005); crypt depth was increased in these last three groups. Muscle Wall Thickness: Control 44.00 μm (±1.30), Botox 44.50 μm (±4.72), SG 49.38 μm (±2.82), and SG+Botox 72.88 μm (±4.15). There was no difference between Control vs Botox. There was a difference between Control vs SG and Botox vs SG (P<0.05). There was a difference between SG vs SG+Botox (P<0.005). The muscle wall thickness was increased in Botox, SG, and SG+Botox. PCNA index: Control 31.88% (±3.27), Botox 41.63% (±5.09), SG 33.88% (±3.64), and SG+Botox 45.13% (±4.08). There were differences between Control vs Botox (P<0.005) and SG vs SG+Botox (P<0.0005). There was no difference between Control vs SG and Botox vs SG+Botox. Botox increased the PCNA index (Figure 4).

### Intestinal transit time (ITT)

**Figure 5:**
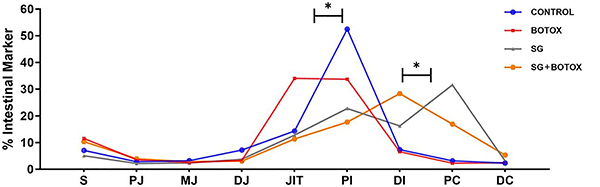
Intestinal transit time from the stomach (S) to the colon (C) amongst the four experimental groups. S: stomach; PJ: proximal jejunum; MJ: middle jejunum, DJ: distal jejunum; JIT: jejunum ileum transition; PI: proximal ileum; DI: distal ileum; PC: proximal colon; and DC: distal colon. *P<0.05 (ANOVA and Tukey's *post hoc* test).

The ITT revealed a significant difference in transit time between the Control and Botox groups, which had intact intestines. Conversely, the SG and SG+Botox groups showed contrasting results with a shorter transit time. The comparison of the toxin's effect was significant in both complete intestines, Control *vs* Botox (P<0.05), and in short intestines, SG *vs* SG+Botox (P<0.05). Notably, the SG+Botox group exhibited a slowing of the ITT near the botulinum toxin application site (JIT), a finding that significantly differed from the SG group (P<0.05) (Figure 5). These findings have significant implications for the treatment of gastrointestinal conditions.

## Discussion

The compensatory process involved in achieving enteral autonomy is intestinal adaptation, characterized by structural and functional changes that compensate for the loss of surface area of the intestinal mucosa and help to increase the absorptive capacity of the remaining intestine. Specific microscopic and morphological changes include a) increased villus and crypt depth, b) proliferation of myocytes and enterocytes, c) elongation and dilation of the small intestinal remnant, d) and decreased enterocyte apoptosis. These changes result in an increased mucosal mass, enlargement of mucous folds, and increased muscle thickness, bowel circumference, and length (15).

In our study, despite the expected adaptation, the histology still identified that the villus height was increased in the Botox and SG+Botox groups (P<0.005). The crypt depth increased in Botox, SG, and SG+Botox (P<0.005); muscle thickness increased in all comparisons except Control *vs* Botox. These results were comparable to Botox results in adult rats and showed histological results compatible with intestinal adaptation at the site where the toxin was administered (12).

Distention of the remnant bowel is the most common consequence of a massive resection. This adaptive gut process has led to the possibility of designing autologous gastrointestinal reconstructive procedures. The prerequisite for successful non-transplant surgery is evidence of a segment or segments of dilated bowel with a lumen diameter greater than twice the normal for age and weight (16).

Our results demonstrated that Botox was efficient in intestinal dilation at the site where it was injected, reaching almost twice the loop dilation after seven days (Figure 3). Furthermore, toxin-induced chemical shutdown in only a small intestinal segment resulted in a prolonged ITT. Regarding the adaptation time of seven days, it seems that with only one segment treated with Botox, it was relevant and sufficient to promote histological changes; seven days of adaptation in the rat is equivalent to 3 to 6 months of human life (17).

Surgical attempts to improve mucosal absorption vary and are imaginative. Supplementation with L-arginine (18), leptin (19), fish oil (13), and melatonin (20), some substances that exist in the gastrointestinal tract and influence mitotic activity applied daily in an experimental model of SG in rats, improved intestinal adaptation. Experimental studies on colonic aganglionosis in mice with chemical denervation of the intestinal segments would potentially delay ITT and improve nutrient absorption (21).

The beneficial effects of Botox application are likely due to a possible reduction in peristalsis, which could facilitate the absorption of nutrients by the intestine. This hypothesis is reinforced by a previous report that the toxin reduces ileal peristalsis by targeting cholinergic neurons, as observed in botulism in the SG of adult rats (12). Our results showed a decrease in ITT in the Botox group. The results were similar to those found in the benzalkonium chloride (BAC) model to treat SG, which showed less transit in groups with a denervated intestinal segment (22). In the same model of SG that we used, the protective factor for ICV was verified; in part, Botox exerted a protective effect against ITT, even without ICV. The main advantage of using the toxin over BAC is that Botox has already been tested for clinical applications and is safe for human patients (23).

It is important to note that in our study, the isolated use of Botox resulted in the elongation of the villi and an increase in the depth of the crypts and ITT, even in animals without short intestines (Figures 4 and 5). Therefore, Botox directly affects the gastrointestinal tract in isolation and not just as a support or as a catalyst for other neuroendocrine phenomena related to intestinal adaptation in intestinal failure secondary to large resections. These results would allow consideration of the use of botulinum toxin in other clinical situations, such as derivations or proximal ostomies, with the possibility of reducing losses and complications related to these situations.

There are results from botulinum toxin application in the sphincter complex of the intestine. Still, there are no reports on histological alterations of the intestine secondary exclusively to its use (24). In children, Botox has been used in achalasia of the internal sphincter (25), constipation, and Hirschsprung disease (26), and in neonates for a variety of indications not approved by the FDA, including congenital muscular torticollis, spastic trismus, and neonatal brachial plexus palsy, and our experimental findings could show benefits in using the toxin for SBS (27).

Botulinum toxin inhibits the exocytotic release of acetylcholine in motor nerve terminals, leading to a decrease in muscle contraction (28). Botox, when injected into the striated muscle, begins its paresis effect normally between the 2nd and 5th day, and this persists for two months when recovery gradually occurs (29). P2X receptors and nicotinic acetylcholine receptors (nAChRs) are ligand-gated cation channels that mediate rapid synaptic excitation in the enteric nervous system (ENS) and help control intestinal motility (30). We believe that the effect of applying the toxin on intestinal smooth muscle can be explained by a similar mechanism that occurs in bladder smooth muscle where Botox promotes a decrease in sub-urothelial P2X3 receptors, relaxing the sphincter muscles and improving the symptoms of the disease (31).

The limitation of our study lies in the amount and the time of Botox administration. We wanted to evaluate the effect of the toxin and obtain a result, but the possibility of applying it in number, quantities, and intestinal distances could have demonstrated greater dilation and perhaps faster intestinal adaptation and weight gain.

In conclusion, in an experimental model of large intestinal resection in recently weaned rats, the application of local Botox close to the anastomotic site increased the diameter of the loop and intestinal transit time. The amount and intestinal distances between Botox applications still need to be tested. However, toxin application could be a plausible surgical strategy for faster intestinal adaptation in neonates with SBS.
